# A comparative analysis of estrogen receptors, ACE2 and cytokines in pre-and postmenopausal women and men with COVID-19

**DOI:** 10.3389/fpubh.2025.1554024

**Published:** 2025-04-28

**Authors:** Mélida Del Rosario Lizarazo-Taborda, Natali Vega-Magaña, Carlos Daniel Díaz-Palomera, Julio César Villegas-Pineda, Marisol Godínez-Rubí, Rubén Alberto Bayardo-González, Adrián Ramírez-de-Arellano, Ana Laura Pereira-Suárez

**Affiliations:** ^1^Maestría en Microbiología Médica, Departamento de Microbiología y Patología, Centro Universitario de Ciencias de la Salud, Universidad de Guadalajara, Guadalajara, Mexico; ^2^Laboratorio de Investigación en Cáncer e Infecciones, Departamento de Microbiología y Patología, Centro Universitario de Ciencias de la Salud, Universidad de Guadalajara, Guadalajara, Mexico; ^3^Departamento de Morfología, Centro Universitario de Ciencias de la Salud, Universidad de Guadalajara, Guadalajara, Mexico; ^4^Laboratorio de Patología Diagnóstica e Inmunohistoquímica, Centro de Investigación y Diagnóstico en Patología, Departamento de Microbiología y Patología, Centro Universitario de Ciencias de la Salud, Universidad de Guadalajara, Guadalajara, Mexico; ^5^Departamento de Clínicas Odontológicas Integrales, Centro Universitario de Ciencias de la Salud, Universidad de Guadalajara, Guadalajara, Mexico

**Keywords:** COVID-19, SARS-CoV-2, ACE2, estrogen receptors, estrogens

## Abstract

The pandemic of Coronavirus Disease 2019 (COVID-19) has a significant impact on older individuals, those with comorbidities, and a bias toward males. Mortality, associated with an exacerbated immune response by proinflammatory cytokines, suggests potential hormonal influences in this scenario. The objective of this research was to analyze the expression of Estrogen Receptor *α* (ERα), Estrogen Receptor *β* (ERβ), G Protein-Coupled Estrogen Receptor (GPER), and the Angiotensin-Converting Enzyme 2 (ACE2) receptor, as well as their relationship with the viral load of SARS-CoV-2 and serum cytokine levels in three demographic groups of unvaccinated individuals diagnosed with COVID-19: premenopausal women, postmenopausal women, and men. The presence and expression of ERα, ERβ, and GPER, along with the ACE2 receptor, were analyzed by immunofluorescence assays in cells obtained from nasopharyngeal swabs of individuals with confirmed COVID-19 through RT-qPCR testing. Additionally, serum cytokine levels were evaluated using a chemiluminescent microparticle immunoassay. The results highlighted notable disparities in the expression of ERα and ACE2, as well as a higher expression of IL-8 and MIP-1β in the premenopausal women group compared to postmenopausal women and men. These findings suggest that in premenopausal women with COVID-19, the elevated expression of ERα and ACE2 could play a protective role, strengthening the antiviral immune response. The importance of exploring the complex hormonal and molecular influences in the pathogenesis of COVID-19 is emphasized, underscoring the need for additional research to better understand the factors determining severity and immune response in different demographic groups.

## 1 Introduction

The Coronavirus Disease 2019 (COVID-19) is a disease caused by a coronavirus known as Severe Acute Respiratory Syndrome Coronavirus 2 (SARS-CoV-2), which is a member of the betacoronavirus family. SARS-CoV-2 is the third betacoronavirus to trigger an epidemiological outbreak, following SARS-CoV in 2002 and MERS-CoV in 2012 (1). SARS-CoV-2 enters host cells by interacting with ACE2, an enzyme central to the Renin-Angiotensin System (RAS) and widely distributed across various tissues (2). The more severe outcomes associated with COVID-19, including elevated mortality rates, have shown a higher prevalence in men. These catastrophic events arise from a combination of factors, including gender-specific behavioral patterns as well as genetic and hormonal influences. Sexual dimorphism, which refers to the biological differences between males and females, also plays a role in immune response and susceptibility to SARS-CoV-2 infection. These biological variations can affect the severity of the disease and highlight the disparities in clinical outcomes (3).

Experimental research using cellular and animal models has provided conclusive evidence regarding the significant influence of sex hormones, particularly estrogens, on susceptibility to viral infections, including those related to COVID-19. In studies conducted with infected transgenic mice, it was observed that males exhibited a greater vulnerability to SARS-CoV infection, resulting in higher mortality rates and more severe symptoms. Conversely, females demonstrated protection associated with estrogen receptor signaling. This protective effect was confirmed through experiments showing increased mortality in females subjected to ovariectomy or treated with an estrogen receptor antagonist (4). These findings emphasize the similarities in gender-based susceptibility between mice and humans, suggesting that estrogen receptor signaling plays a crucial role in female defense against SARS-CoV. Like humans, these results highlight how hormonal differences can influence immune response and the severity of infection. However, it is essential to remember that these studies have been conducted in animal models, which limits the direct extrapolation to the human population. This underscores the importance of conducting additional research to validate these observations and fully understand their clinical relevance.

Recent studies show that 17β-estradiol reduces the expression of ACE2 and TMPRSS2, key genes involved in SARS-CoV-2 entry into lung epithelial cells. These results suggest a potential protective role of estrogen against severe COVID-19 infections, especially in women, as higher levels of estrogens in this group may modulate the expression of these critical genes involved in viral entry and contribute to a more effective immune response (5).

Estrogen receptors alpha (ERα) and beta (ERβ) are fundamental in regulating various biological functions, including the immune response and the modulation of inflammatory processes. In particular, the ERβ has been shown to be present in multiple tissues and plays a critical role in mediating the effects of estrogens. This finding raises important questions about the role of sex hormones, including estrogens, and their receptors in mediating these effects in the context of COVID-19 (6). A specific interaction between ERα and a particular motif in the S2 subunit of the viral S protein has been reported, highlighting the direct influence of ERα on molecular events regulating S protein activity. This discovery not only reveals an innovative connection in cellular biology but also suggests a possible implication of the ERα in cellular responses to viral infection (7). Subsequently, evidence was presented suggesting that this interaction between the S protein and ERα could contribute to the activation of the coagulation cascade, leading to severe coagulation syndrome in various organs, a potentially life-threatening condition associated with acute SARS-CoV-2 infection (8). Despite these findings, the involvement of ERs in SARS-CoV-2 infection remains an active area of research. Our objective was to examine the divergences in the expression of ERα, ERβ, and GPER, ACE2, and cytokines through an analysis that considers the biological and hormonal differences in specific groups, including premenopausal women, postmenopausal women, and men with COVID-19.

## 2 Materials and methods

### 2.1 Studied population

Between April and October 2021, blood and nasopharyngeal swab samples were collected at the Laboratory of Emerging and Re-emerging Diseases (LaDEER), affiliated with the Centro Universitario de Ciencias de la Salud (CUCS) at the Universidad de Guadalajara (UdeG). A 5 mL peripheral blood sample was collected from each participant using a BD Vacutainer tube (Cat. No. BD-368175, Becton, Dickinson and Company, NJ, USA) containing a coagulation activator. The sample was centrifuged at 3,500 rpm for 10 min to obtain the serum, which was then aliquoted and stored at −80°C until use. The study population comprised 45 participants who presented for COVID-19 diagnosis. Only those who tested positive for SARS-CoV-2 and were unvaccinated against SARS-CoV-2 were included, with the aim of avoiding potential biases arising from vaccination that could interfere with the assessment of the protective influence of estrogen. The participants were assigned to three groups: premenopausal women (*n* = 15), postmenopausal women (*n* = 15), and men (*n* = 15). All participants were over 18 years old; premenopausal women had regular menstrual cycles, while postmenopausal women presented amenorrhea, confirmed by the determination of 17β-estradiol levels, and had not menstruated for over a year without receiving estrogen replacement therapy. Individuals with Human Immunodeficiency Virus (HIV), Hepatitis B Virus (HBV), and Hepatitis C Virus (HCV), as well as those with cancer, smokers, individuals undergoing antibiotic or antiviral treatment, and those receiving hormonal therapy, were excluded from the study. To ensure compliance with the ethical and safety standards, the study received approval from the Ethics and Biosafety Committees of CUCS-UdeG, with protocol number CI-03321.

### 2.2 Diagnosis of participants by qRT-PCR

To determine infection with SARS-CoV-2, RT-qPCR was performed using the CoviFlu Multiplex kit (GENES2LIFE, Cat. No. G2LcoFM-04, Guanajuato, México) in nasopharyngeal swabs. This kit allows for the simultaneous detection of the N2 gene of the SARS-CoV-2 nucleocapsid, as well as Influenza A and B viruses, in addition to a control gene for RNA extraction (RNAseP). The process was carried out following the manufacturer’s instructions. The reactions were processed in the QS5 thermocycler (Thermo Scientific, MA, USA) with the following conditions: 55°C for 10 min, 92°C for 2 min, and 40 cycles of 95°C for 10 s and 60°C for 30 s. The results were analyzed using the real-time PCR system software QuantStudio5 (Thermo Scientific, MA, USA).

### 2.3 Chemiluminescent microparticle immunoassay and cytokine ELISA in serum samples

To determine the serum 17β-estradiol concentration of the three groups of participants was carried out the chemiluminescent microparticle immunoassay technology (Cat. No. B7K720, Abbott, IL, USA). The assay was conducted according to the supplier’s specifications, utilizing the automated ARCHITECT®i1000SR equipment. Cytokine quantification was performed on serum samples from COVID-19 participants using the high-sensitivity multiplex ELISA technique with the commercial Bio-Plex Pro Human Cytokine 17-plex Assay kit (Cat. No. M5000031YV, Bio-Rad Laboratories, CA, USA). This method enabled the simultaneous measurement of G-CSF, GM-CSF, IFN-*γ*, IL-1β, IL-2, IL-4, IL-5, IL-6, IL-7, IL-8, IL-10, IL-12, IL-13, IL-17A, MCP-1, MIP-1β, and TNF-*α*. The process involved adding paramagnetic beads to each well, followed by washing, and the addition of standard dilutions, controls, blanks, and serum samples. After a 2-h incubation, the wells were washed, the antibody detection preparation was added, incubated, and washed again. Finally, Streptavidin-Phycoerythrin was added, washed, and the sample was resuspended for analysis using the Bio-Plex MAGPIX system.

### 2.4 Immunofluorescence and quenching of autofluorescence in nasopharyngeal cells

To evaluate the presence of ERα, ERβ, GPER, and ACE2, immunofluorescence assays were conducted on nasopharyngeal epithelial cells obtained from swab samples and preserved in viral transport medium. A 100 μL sample was taken and centrifuged at 2000 rpm for 2 min using the Sigma 2–7 Cyto system (Sigma Laborzentrifugen GmbH, Göttingen, Germany). After centrifugation, the monolayer of extended cells was obtained, and subsequently fixed with acetone for 5 min. The samples were then washed four times: once with PBS-T and three times with PBS (1 mL of solution for each wash).

The samples were permeabilized with 0.2% Tween 20 in PBS and incubated for 10 min. Following this, a wash was performed, and blocking was carried out using a solution of 10% fetal bovine serum and 1% bovine serum albumin (BSA) in PBS, and the samples were incubated at 37°C for 1 h. Subsequently, the primary antibody solution was added with the following dilutions: anti-ERα 1:100 (Cat. No. sc-8002), anti-ERβ 1:50 (Cat. No. sc-373853, Santa Cruz Biotechnology, CA, USA), anti-GPER 1:200 (Cat. No. ab39742), and anti-ACE2 1:100 (Cat. No. ab272500, Abcam, Cambridge, United Kingdom). The samples were incubated overnight in a humid chamber. The next day, washes were performed, and the slides were incubated with the secondary antibody anti-rabbit Alexa 488 (Cat. No. A-11008), anti-rabbit Alexa 594 (Cat. No. A-11012, Invitrogen, CA, USA), or anti-mouse FITC (Cat. No. ab6785, Abcam, Cambridge, United Kingdom), at a dilution of 1:1000, as appropriate, for 2 h in the dark. After this time, another wash was conducted. As an additional step, the solution from the Vector® TrueVIEW® Autofluorescence Quenching Kit (Cat. No. SP-8400-15, Vector Laboratories, CA, USA) was added to each slide for 5 min to eliminate autofluorescence, prepared according to the manufacturer’s instructions. Next, another cycle of washes was performed, and the nuclei were stained with DAPI at a dilution of 1:10000 (Cat. No. D1306, Invitrogen, CA, USA), applying the reagent to each slide for 5 min at room temperature. Subsequently, the same washing procedure was carried out: one with PBS-T, three with PBS, and an additional wash with distilled water. The process concluded by adding VECTASHIELD Vibrance® Antifade Mounting Medium (Cat. No. H-1700, Vector Laboratories, CA, USA) to each slide, followed by sealing with clear nail polish. The images were obtained using a Carl Zeiss LSM 900 fluorescence microscope (Carl Zeiss AG, Göttingen, Germany). At least 5 microscopic fields were captured for each slide in this analysis. The images were analyzed using ImageJ software V:2.14.0/1.54f (National Institutes of Health, MD, USA).

### 2.5 Statistical analysis

Normality analyses were conducted using the D’Agostino and Pearson, Shapiro–Wilk, and Kolmogorov–Smirnov tests. To assess differences between groups in 17β-estradiol, viral load, ACE2, estrogen receptors, and cytokines, Kruskal-Wallis tests were performed, with Dunn’s test used as a *post hoc* analysis. Statistical analysis and graphical representation were carried out using GraphPad Prism software v. 9.0 (GraphPad Software, Inc., CA, USA).

## 3 Results

### 3.1 Sociodemographic and clinical characteristics

A total of 45 participants were included in the study and categorized into three distinct groups: premenopausal women (*n* = 15), postmenopausal women (*n* = 15), and men (*n* = 15). Sociodemographic and clinical information is detailed in Table 1. Significant disparities were observed in the participants, age (*p* < 0.0001), weight (*p* = 0.0009), and height (*p* < 0.0001). Analysis of comorbidities, body mass index and SARS-CoV-2 viral load did not reveal significant differences (Table 1).

**Table 1:** Sociodemographic and clinical data.

Variables	Premenopausal women *n* = 15	Postmenopausal women *n* = 15	Men *n* = 15
Age (years)	35 ± 6.4	58 ± 10.8	36.4 ± 11.4
Weight (kg)	74 ± 17	65.9 ± 14.6	89.6 ± 18
Height (m)	1.61 ± 0.05	1.54 ± 0.04	1.75 ± 0.06
Comorbidities			
Yes (%)	6 (40)	10 (66.7)	9 (60)
No (%)	9 (60)	5 (33.3)	6 (40)
Hypertension (%)	–	2 (13.3)	2 (13.3)
Type 2 Diabetes Mellitus (%)	1 (6.7)	3 (20)	1 (6.7)
Obesity (%)	5 (33.3)	5 (33.3)	6 (40)
Body mass index			
Normal weight (%)	5 (33.3)	3 (20)	8 (53.3)
Overweight (%)	5 (33.3)	6 (40)	1 (6.7)
Obesity (%)	5 (33.3)	5 (33.3)	6 (40)
Malnutrition grade I (%)	–	1 (6.7)	–
SARS-CoV-2			
Viral load (mean, copies/mL)	6.69×10^5^	3.34×10^6^	2.23×10^6^
High (>100,000 copies/mL) (%)	10 (66.7)	10 (66.7)	11 (73.4)
Moderate (10,000–100,000 copies/mL) (%)	3 (20)	1 (6.7)	2 (13.3)
Low (<10,000 copies/mL) (%)	2 (13.3)	4 (26.6)	2 (13.3)

Characteristics of the participants analyzed by groups. Variables include age, weight, height, comorbidities, and viral load.

**Figure 1:**
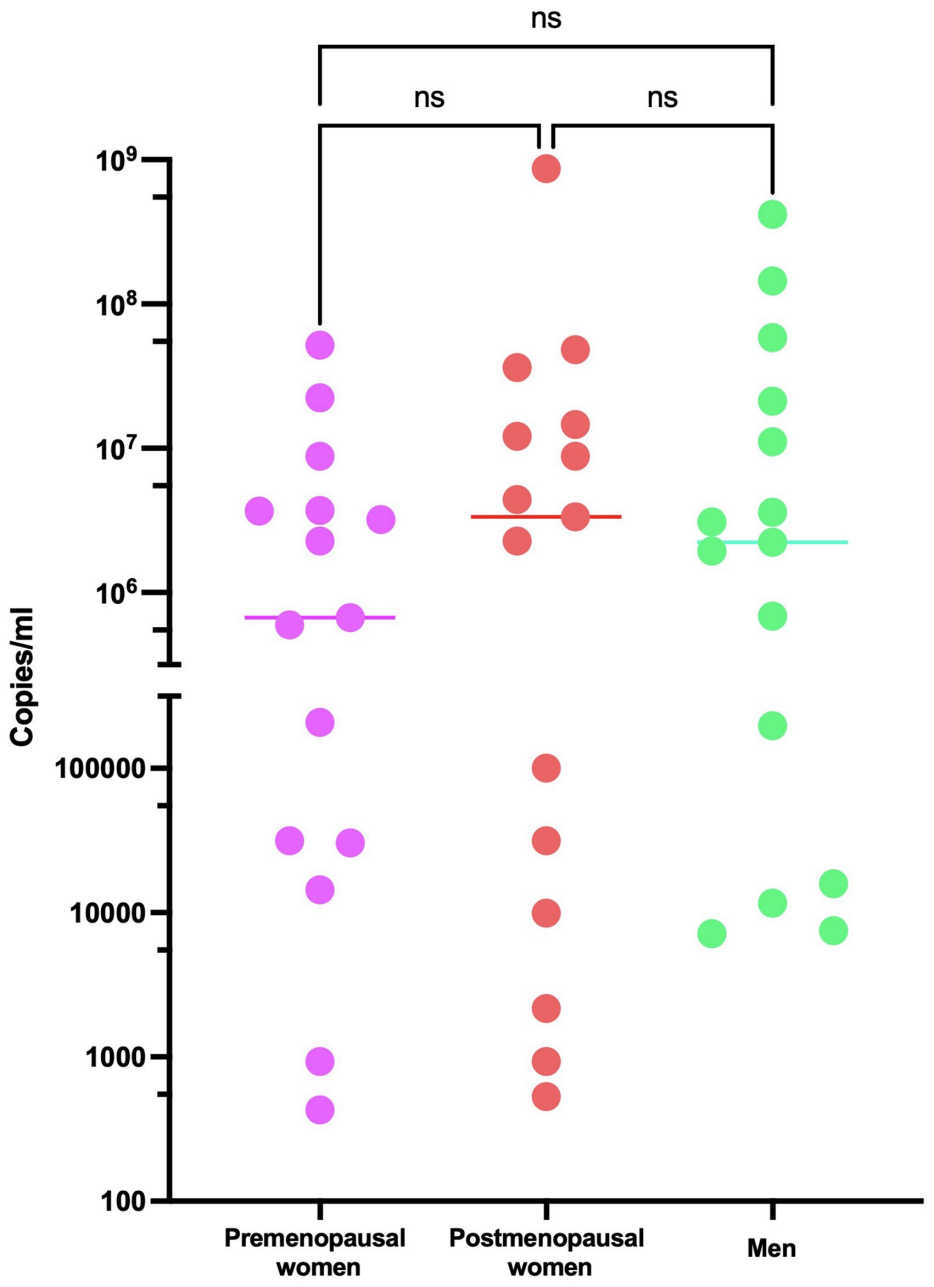
Viral load of SARS-CoV-2 in COVID-19 participants. RT-qPCR assays were performed for the diagnosis of the three studied groups: premenopausal women (*n* = 15), postmenopausal women (*n* = 15), and men (*n* = 15). The horizontal bars indicate the mean of the obtained values, ns: no statistical difference.

**Figure 2:**
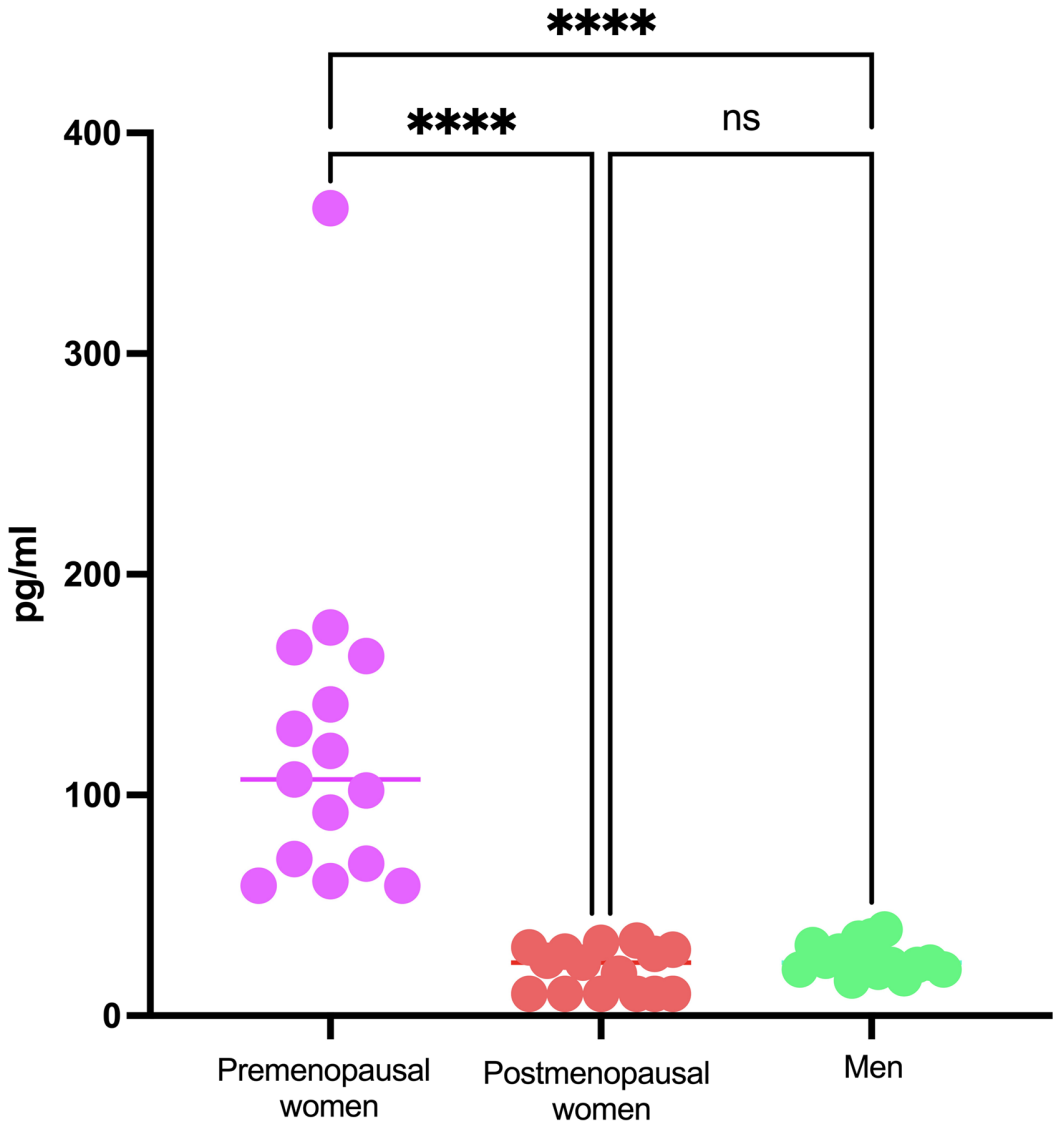
E2 concentration in COVID-19 participants´ serum. Estradiol concentration in premenopausal women (*n* = 15), postmenopausal women (*n* = 15), and men (*n* = 15). Horizontal bars indicate the mean of the obtained values. **** = *p* < 0.0001, ns: no statistical difference.

Regarding the analysis of viral load in the study participants, it demonstrates consistent behavior among the three investigated groups, with no statistically significant differences observed (Figure 1). The levels of 17β-estradiol revealed an appropriate categorization of the groups concerning this hormone and menopausal status. As expected, premenopausal women exhibited higher levels of 17β-estradiol than postmenopausal women and men (Figure 2).

### 3.2 ACE2 is overexpressed in nasopharyngeal cells of premenopausal women

**Figure 3:**
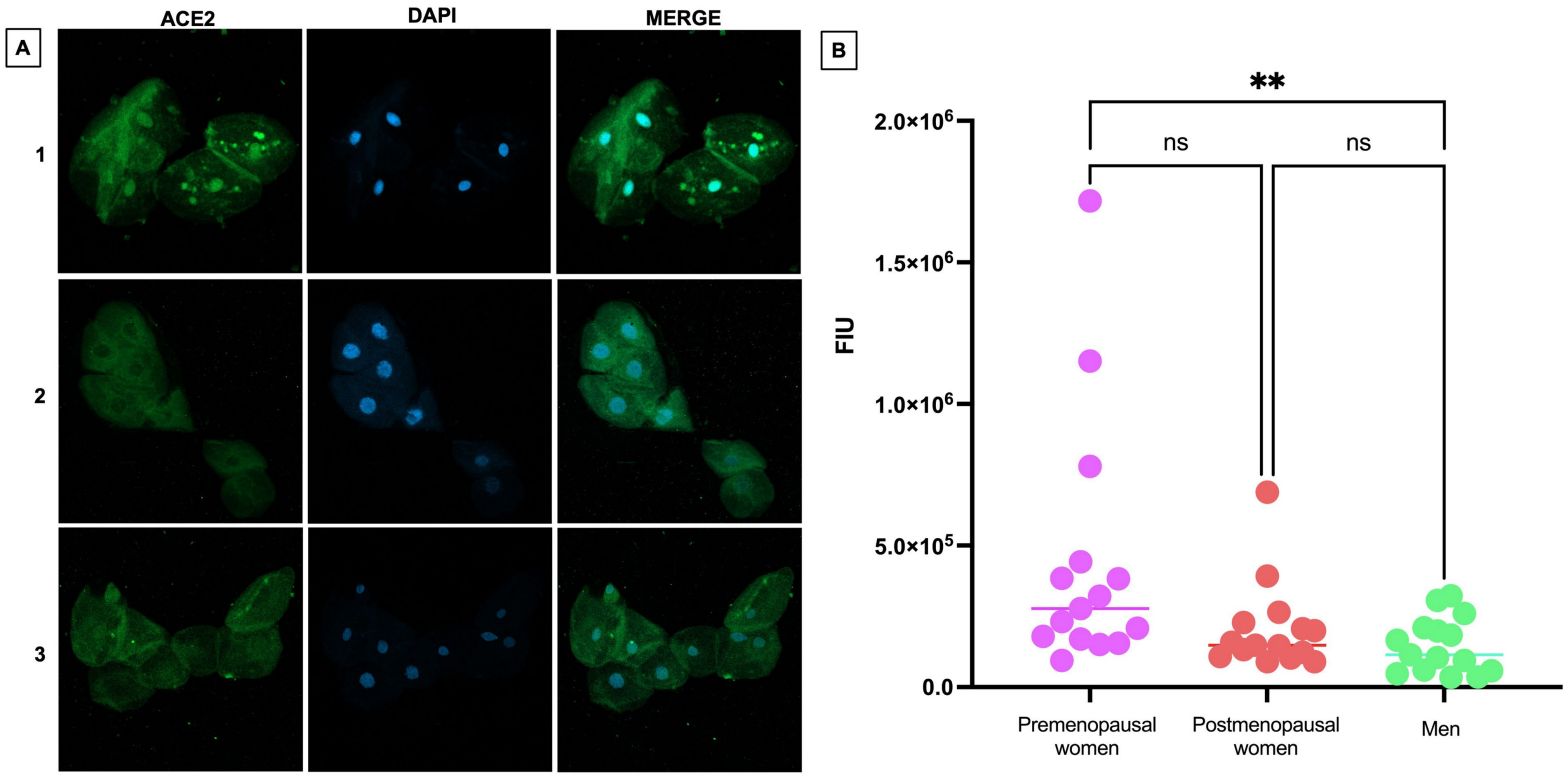
ACE2 expression in nasopharyngeal swab samples from COVID-19 participants. **(A)** (1) premenopausal women (*n* = 15), (2) postmenopausal women (*n* = 15), and (3) men (*n* = 15). In the first column the expression of ACE2 is observed in green, in the second column in blue, nuclei marked with DAPI can be visualized, and in the third column, the merged views at 40X magnification. **(B)** Analysis of ACE2 fluorescence intensity units (FIU). Horizontal bars indicate the mean of the obtained values. ** = *p* < 0.01, ns: no statistical difference.

The expression of ACE2 was investigated in nasopharyngeal epithelial cells obtained by swabbing in the three study groups through immunofluorescence. Representative images showing the distribution of ACE2 are presented, highlighting the presence of the protein in the cytoplasm (Figure 3A). Additionally, a significantly more intense expression was observed in premenopausal participants compared to men (*p* < 0.01). This observation is consistently supported by the quantitative analysis of fluorescence intensity in each group (Figure 3B).

### 3.3 ERα is overexpressed in nasopharyngeal cells of premenopausal women, while ERβ and GPER were expressed indifferently in the 3 groups of participants

**Figure 4:**
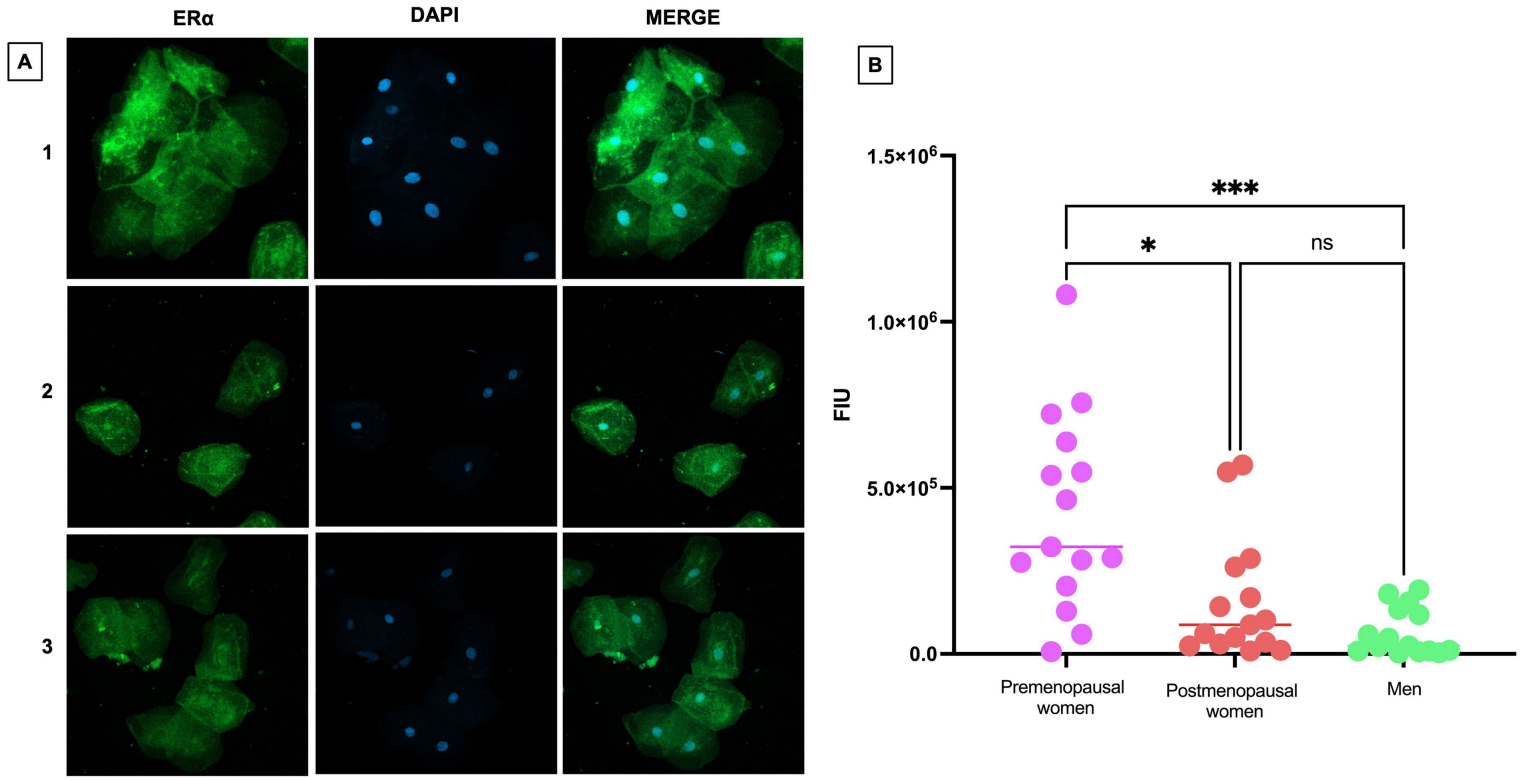
ERα expression in nasopharyngeal swab samples from COVID-19 participants. **(A)** (1) premenopausal women (*n* = 15), (2) postmenopausal women (*n* = 15), and (3) men (*n* = 15). In the first column the expression of ERα is observed in green, in the second column in blue, nuclei marked with DAPI can be visualized, and in the third column, the merged views a 40X magnification. **(B)** Analysis of ERα fluorescence intensity units (FIU). Horizontal bars indicate the mean of the obtained values. * = *p* < 0.05, *** = *p* < 0.001, ns: no statistical difference.

To assess the expression and localization of ERα in nasopharyngeal swab samples from premenopausal women, postmenopausal women and men, immunofluorescence assays were performed (Figure 4A). A differential expression among the studied groups was observed, with higher ERα expression in premenopausal women compared to postmenopausal women (*p* < 0.05) and men (*p* < 0.001). However, no statistically significant differences between postmenopausal women and men (Figure 4B).

ERα is expressed both in the cytoplasm and in the nucleus of the cells. In premenopausal women with COVID-19, ERα was predominantly localized in the cytoplasm, unlike its nuclear localization in postmenopausal women and men.

**Figure 5:**
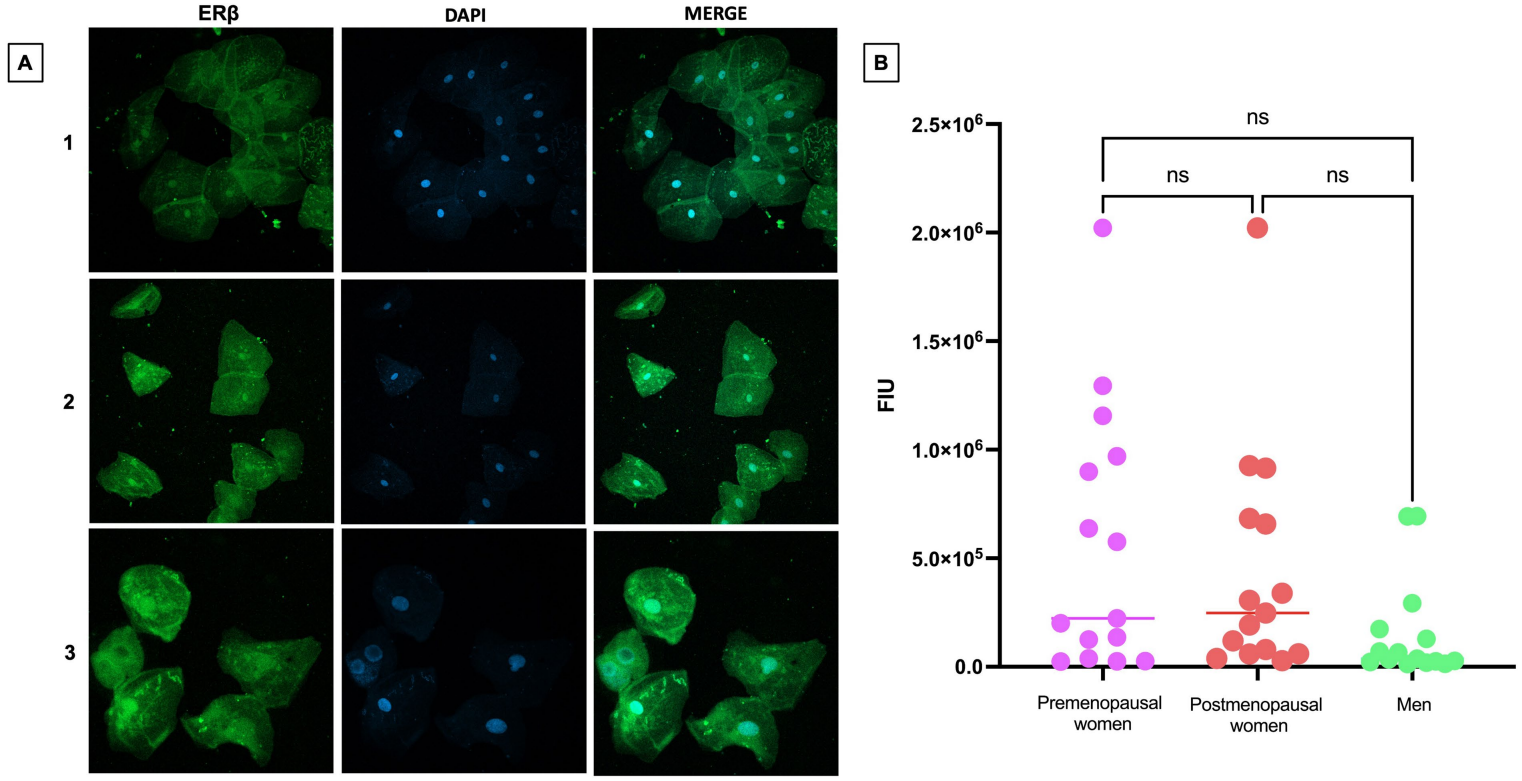
ERβ expression in nasopharyngeal swab samples from COVID-19 participants. **(A)** (1) premenopausal women (*n* = 15), (2) postmenopausal women (*n* = 15) and (3) men (*n* = 15). In the first column the expression of ERβ is observed in green, in the second column in blue, nuclei marked with DAPI can be visualized, and in the third column, the merged views at 40X magnification. **(B)** Analysis of ERβ fluorescence intensity units (FIU). Horizontal bars indicate the mean of the obtained values. ns: no statistical difference.

The expression levels of ERβ were observed to be abundant in both the cellular nuclei and the cytoplasm (Figure 5A). This expression pattern remained consistent and uniform across the three studied groups, suggesting a stable regulation of ERβ under the different analyzed conditions. The expression levels in the studied groups show that there are no statistically significant differences in ERβ expression between premenopausal women, postmenopausal women, and men (Figure 5B). These findings are supported by a statistical analysis that confirms the homogeneity of expression across the different groups.

**Figure 6:**
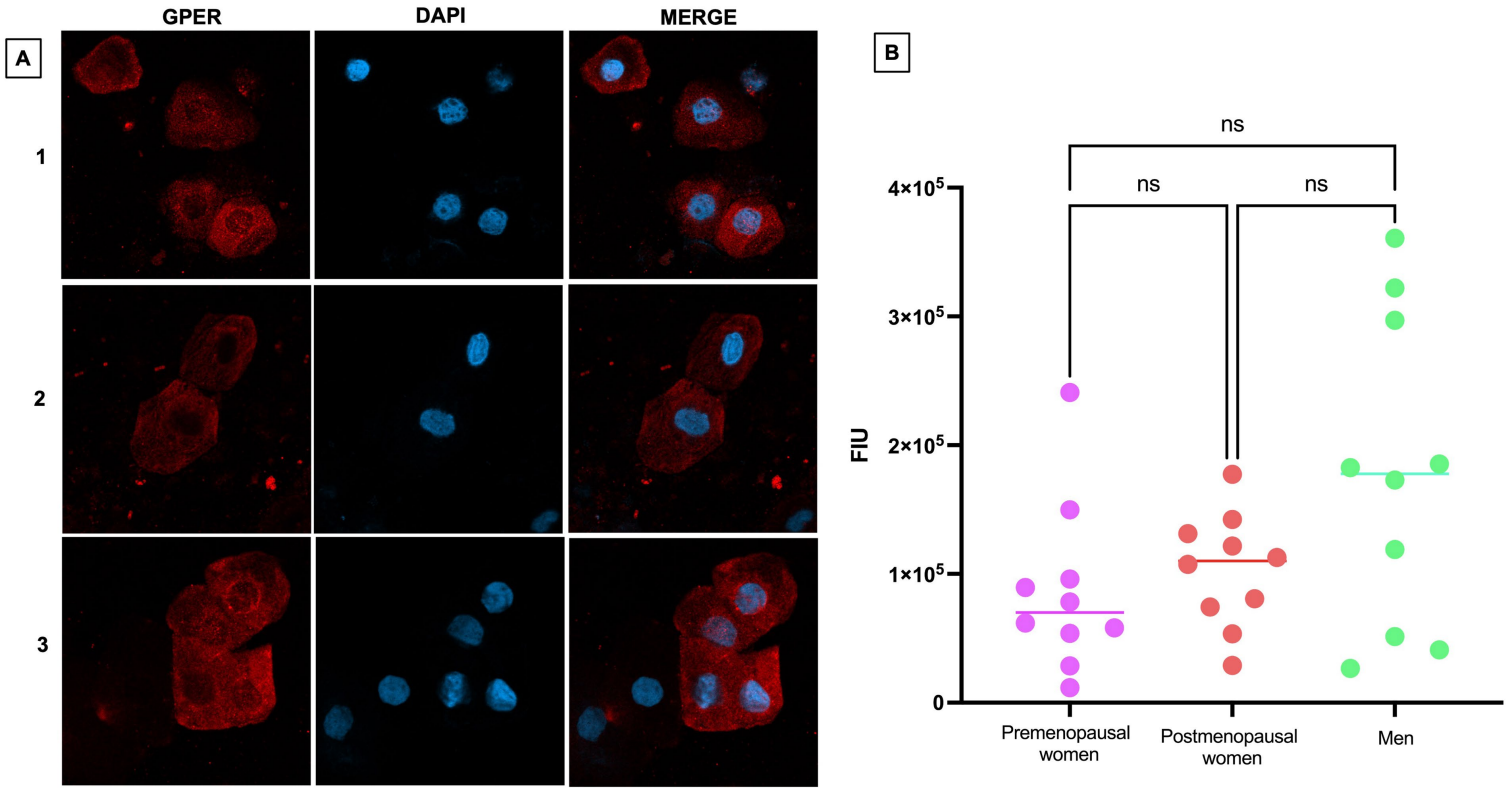
GPER expression in nasopharyngeal swab samples from COVID-19 participants. **(A)** (1) premenopausal women (*n* = 10), (2) postmenopausal women (*n* = 10), and (3) men (*n* = 10). In the first column the expression of GPER is observed in red, in the second column in blue, nuclei marked with DAPI can be visualized, and in the third column, the merged views at 40X magnification. **(B)** Analysis of GPER fluorescence intensity units (FIU). Horizontal bars indicate the mean of the obtained values. ns: no statistical difference.

Regarding the expression of GPER, a weak expression of this receptor was observed, both in the cell membrane and in the cytoplasm (Figure 6A). Although no statistically significant differences were found between the different groups, there appears to be a trend toward higher expression in the male group compared to premenopausal and postmenopausal women (Figure 6B).

### 3.4 Cytokine analysis in serum

**Figure 7:**
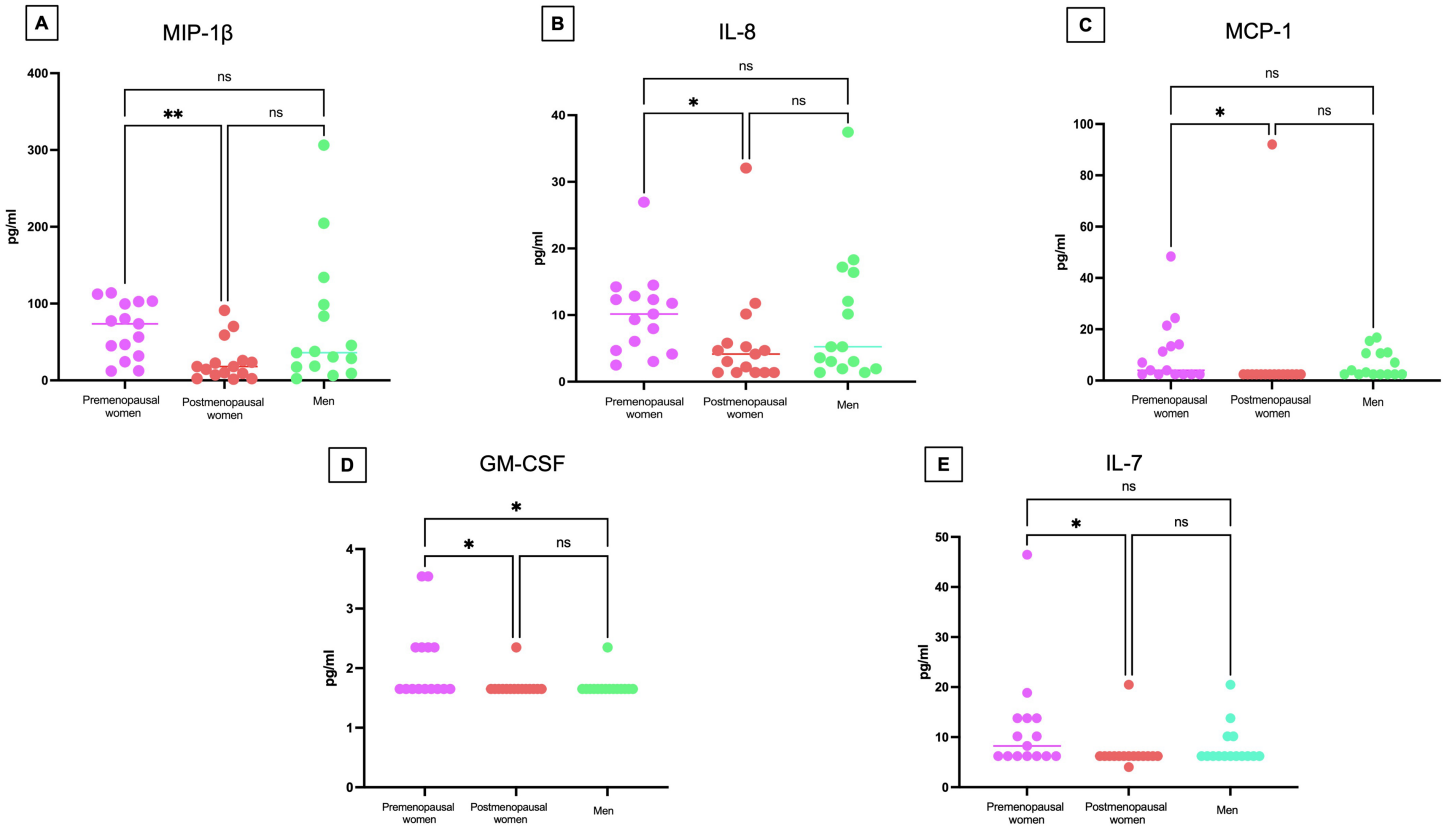
Cytokine concentrations in COVID-19 participants´ serum. Comparison of the concentrations of **(A)** MIP-1β, **(B)** IL-8 **(C)** MCP-1 **(D)** GM-CSF, and **(E)** IL-7 in pg/mL among premenopausal women (*n* = 15), postmenopausal women (*n* = 15), and men (*n* = 15). Each point represents a study subject. The horizontal bars indicate the mean of the obtained values. * = *p* < 0.05, ** = *p* < 0.01, ns: no statistical difference.

Cytokine levels in serum were measured to assess the inflammatory response (Figure 7). Significant differences were observed in several cytokines, including MIP-1β (*p* < 0.01) (Figure 7A), IL-8 (*p* < 0.05) (Figure 7B), MCP-1 (*p* < 0.05) (Figure 7C), GM-CSF (*p* < 0.05) (Figure 7D), and IL-7 (*p* < 0.05) (Figure 7E), with higher levels found in the premenopausal women group compared to the postmenopausal women. Additionally, GM-CSF (*p* < 0.05) also exhibited a statistically significant difference between premenopausal women and men.

Additional cytokines from the panel were not considered, as their concentration in many of the analyzed samples did not reach the minimum detection limit. The results suggest that premenopausal women had higher cytokine levels, reflecting a more active immune response.

## 4 Discussion

In SARS-CoV-2 infection, a higher severity and mortality have been observed in men than in women (9–12). This sex-based difference in disease severity may be related to hormonal disparities between genders, particularly to estradiol concentration, and to the expression of estrogen receptors, such as ERα, which has been suggested as a potential protective factor against viral infection and inflammatory response (13). Estrogens exert protective effects by regulating the RAS. In this context, the interaction of estradiol with the ERα activates the unconventional RAS leading to an increase in the generation of angiotensin 1–7 (14, 15). This phenomenon becomes particularly relevant in the context of COVID-19, as the modulation of the RAS, linked to the expression of ACE2, could have implications for disease protection, highlighting the interconnection between estrogens and molecular mechanisms that may influence susceptibility and severity of virus infection. This is underscored by a study comparing women over 50 years of age, who received estradiol therapy to those who did not, revealing a significant reduction in the risk of COVID-19 mortality in the hormone therapy group, suggestive of the protective effect of estradiol (16).

Our study corroborates that the divergence in 17β-estradiol concentration is not limited to gender but also varies according to reproductive age. We observed high levels of estradiol in premenopausal women and low levels in postmenopausal women and men. Paradoxically, these last two groups have been the most affected by COVID-19 (17–20). Unexpectedly, we did not observe a differential behavior in viral load between groups that could indicate any relationship with estrogen concentration, while Lemes et al. (21), in a study using 17β-estradiol-stimulated Vero E6 cells, indicated an *in vitro* reduction in the viral load of SARS-CoV-2 attributed to the hormonal effect. It is crucial to note that disparities in the findings could be attributed to differences in experimental conditions; while the previously mentioned study was conducted under controlled experimental conditions using a cell line, our work involved individuals, where variables such as the timing of inoculation and initial viral dose were not under our control (21). Literature emphasizes the importance of considering factors such as timing of inoculation, due to the initial exposure dose to the virus can influence the severity and progression of the disease. Viral replication tends to peak in the early days after infection and then decreases. It has been observed that viral load is usually higher shortly before or at the onset of symptoms, decreasing as the infection progresses (22, 23). On the other hand, in addition to the timing of inoculation and initial viral dose, individual and clinical factors such as age, host immune response, and the presence of comorbidities can also influence viral load and the severity of the disease (24–26).

Our most comprehensive approach highlights the complexity of viral dynamics in the clinical context, underscoring the need for caution when interpreting *in vitro* results in the clinical setting. In particular, our study has demonstrated the variation of ACE2 expression in cells obtained from nasopharyngeal swabs of COVID-19 individuals who have not received vaccines against SARS-CoV-2, analyzed using the immunofluorescence technique and from a gender and hormonal perspective. To date, no published research has addressed this specific approach, making these findings especially relevant. It has been observed that premenopausal women have significantly higher ACE2 expression compared to men. It is worth noting that ACE2 expression shows notable variability depending on the type of tissue or cell analyzed. In this sense, contrasting research has been found regarding the expression of ACE2 in lung tissue, where some reports indicate a higher expression of ACE2 in the male population and others in females. Therefore, considering these variations is essential to better understand individual susceptibility to the virus (27–29).

In this context, the discovery of increased expression of ACE2 in cells of the nasopharyngeal epithelium of premenopausal women, compared to men, suggests a possible correlation with higher concentrations of 17β-estradiol and the elevated expression of ERα. The directly proportional relationship between ACE2, 17β-estradiol and ERα reinforces the idea that sex hormones play a significant role in ACE2 expression. This study not only highlights the variability of ACE2 expression in different tissue contexts but also suggests that sex hormones and their receptors may influence this expression. These findings highlight the need to consider hormonal factors in the body’s response to SARS-CoV-2 and raise questions for future research into personalized therapeutic approaches.

The observation of a higher expression of ERα in nasopharyngeal epithelial cells of premenopausal women, compared to postmenopausal women and men, suggests a potential connection with the regenerative capacity of estrogens. It is noteworthy that this analysis was based on cells obtained from nasopharyngeal swabs, providing a direct perspective of the cellular environment at the specific site of viral infection. Activation of ERα has been associated with regenerative processes, including alveolar regeneration (30), raising the intriguing possibility that the increased expression of ERα in premenopausal women could contribute to a cellular environment conducive to regeneration. This phenomenon may have relevant implications for the health of the nasopharyngeal epithelium, particularly considering the context of SARS-CoV-2 infection. However, further studies are needed to fully understand the interaction between ERα expression and nasopharyngeal epithelial cells regeneration in this specific context. ERα plays a crucial role in regulating the expression and activity of ACE2 in various tissues, including the cardiac tissue (31), human atrium (32) and pulmonary tissue of mice infected with SARS-CoV-2 (33).

In our study, we found a higher expression of ERα in cells obtained from nasopharyngeal swabs of premenopausal participants compared to postmenopausal participants and men with COVID-19. The presence of high estrogen levels in premenopausal women, along with the increased expression of ERα, is associated with greater protection of organs against the adverse effects of infection with SARS-CoV-2. The correlation between ERα and ACE2 suggests that pathways associated with estrogen may play a crucial role in this protection. A recent study using a male and female mouse model has demonstrated that females exhibit higher levels of ACE2 and ERα expression, which translates to greater resistance to severe infections (33, 34). The interaction between estrogens and ACE2, as well as the positive regulation of ERα in the presence of estrogens, is proposed as a protective mechanism in women. Notably, previous research has reported that 17β-estradiol can promote the cleavage of the soluble fraction of ACE2 in Vero cells, leading to its secretion into the extracellular environment, where it binds to SARS-CoV-2, potentially inhibiting its infective capacity (35). This result aligns with our findings, suggesting that the enhanced immune response observed in premenopausal women against COVID-19 could be orchestrated by 17β-estradiol. This observation raises the possibility of exploring therapies that modulate estrogen pathways in men to enhance the immune response and reduce disease severity.

In the localization of ERα assessed by immunofluorescence, we observed that the expression of this receptor appears to be more pronounced in the cytoplasm compared to the cell nucleus (Figure 4A). This finding is consistent with recent reports indicating that the SARS-CoV-2 spike protein directly interacts with ERα in human cells, suggesting that this interaction could induce a change in the localization of ERα within the cell, leading to its accumulation primarily in the cytoplasm instead of its usual position in the cell nucleus (7). The altered localization of ERα may impact transcription and other cellular responses, including cytokine and growth factor regulation, which are critical for immune response and inflammation. This provides a new perspective on how the virus could impact cellular functions through its interaction with hormonal receptors.

Referring to ERβ, its presence has been documented in a variety of tissues and systems, including the ovary, cardiovascular system, lungs, male reproductive organs, kidney, and the immune system, among others (36). Our study shows the behavior of ERβ expression in nasopharyngeal epithelial cells of individuals with COVID-19, although no significant differences in expression levels were observed when comparing premenopausal women, postmenopausal women, and men, this result indicates the stability of ERβ expression in individuals with SARS-CoV-2 infection. It also emphasizes that, despite the simultaneous co-expression of ERα and ERβ in the same tissue, the proportion of expression of both receptors may vary in specific tissues, allowing for the modulation of their distinct functions. In this context, it is essential to discern the balance of expression between ERα and ERβ, as these receptors play unique and characteristic roles (36).

The GPER has been less studied, in part due to its recent discovery (37). Overall, it has been associated with various pathologies such as coronary diseases (38), kidney disease (39), metabolic diseases (40), among others. GPER has been reported to be crucial for suppressing IFN-*γ* signaling during pregnancy, especially when female mice are infected with influenza A virus, thus protecting the fetus during maternal infections (41). No direct studies have compared GPER expression between men and women, since this receptor is mainly studied in the context of the female reproductive system. Clinical data indicate that men, following SARS-CoV-2 infection, tend to experience higher levels of hospitalization and mortality from COVID-19 compared to women; the involvement of GPER in this disparity has been suggested, since its activation, demonstrated in experimental models, reduces the viral load of SARS-CoV-2 in bronchial cells infected with SARS-CoV-2 (42). Pharmacological antagonism of GPER increased the viral load, suggesting an important role of this receptor in COVID-19 (42). GPER was expressed in nasopharyngeal epithelial cells, with no statistically significant differences observed between groups. However, there is a trend of higher expression in the group of men compared to premenopausal and postmenopausal women. Considering the additional reference of the correlational analysis between this marker and viral load, we did not obtain a similar result with what has been reported *in vitro*. Therefore, it would be interesting to expand studies to understand the behavior of this receptor in the context of COVID-19 in a larger cohort of individuals from a sex difference perspective and to include individuals with greater severity of COVID-19.

In our outpatient study during the COVID-19 pandemic, we have observed a significantly higher expression of the cytokines MIP-1β, IL-8, MCP1, GM-CSF, and IL-7 in the premenopausal women group compared to the postmenopausal women group. These results suggest a more robust immune response in premenopausal women at the onset of infection, possibly related to a higher estrogen concentration in this group. Unlike most studies focusing on critically ill patients, our focus on outpatients provides a valuable perspective to understand the variability of immune response in different patient profiles.

Supporting our research, previous studies have highlighted the importance of IL-8 and MIP-1β in relation to the severity of the disease in COVID-19 individuals (43–46). IL-8, known for its role in neutrophil attraction and involvement in inflammatory lung diseases, has shown elevated concentrations in patients with more critical outcomes. Similarly, MIP-1β, essential in recruiting various cells of the immune system, has also demonstrated its association with the severity of the disease (43, 44). A significant disparity in GM-CSF cytokine levels has been observed between premenopausal women and men in our study on COVID-19 individuals. This finding underscores the critical importance of GM-CSF cytokine in the immune response to COVID-19 infection. Additionally, the detection of higher expression of ERα in premenopausal women reinforces the relevance of hormonal signaling in immune regulation. Signaling through ERα may enhance the inflammatory pathway of GM-CSF-mediated dendritic cell (DC) differentiation during inflammatory states (47). This process may lead to the development of DCs with improved functionality, further emphasizing the importance of the interaction between estrogen receptors and GM-CSF cytokine in the immune response against COVID-19.

This molecular context suggests a potential correlation between elevated GM-CSF cytokine levels, increased expression of ERα, and a more effective immune response, especially in premenopausal women. This robust immune response in premenopausal women could have crucial implications for the successful resolution of COVID-19 infection, highlighting the importance of considering gender differences in disease severity and progression.

The MCP-1, an inflammatory cytokine, was found at higher levels in premenopausal women compared to postmenopausal women in our study. This finding suggests a potentially earlier and more vigorous activation of the immune system in premenopausal women. Although MCP-1 has been associated with disease severity and mortality risk during the COVID-19 pandemic, it is crucial to consider that our study focused on outpatient, indicating a less advanced stage of the disease. In this context, elevated levels of MCP-1 could indicate a more effective and potentially protective immune response, regardless of disease severity (3). This phenomenon suggests a possible duality in the role of MCP-1 in COVID-19 pathogenesis: it could act as a severity biomarker in critical cases, but also as an indicator of an effective immune response in early stages of the disease. These observations underscore the need for further investigation into the role of MCP-1 in different stages of the disease and in different populations, including hormonal considerations, to fully understand its involvement in the immune response to COVID-19.

Our study also revealed an intriguing discrepancy: while IL-7 levels were higher in premenopausal women compared to postmenopausal women, no statistically significant differences were observed between women and men. This contrasts with the findings of Chi et al. (48), who reported higher IL-7 levels in male outpatients compared to female outpatients. The difference in IL-7 concentrations between premenopausal and postmenopausal women may be influenced by hormonal fluctuations, particularly variations in 17β-estradiol levels. These hormonal changes could regulate the expression and modulation of cytokines such as IL-7, reinforcing the hypothesis that estrogens play a protective role in the female immune response.

In contrast, the findings of Chi et al. (48) suggest that in men, an increased production of certain cytokines, including IL-7, may contribute to both pathogen clearance and excessive inflammatory responses. This inflammatory response could explain why some men are more susceptible to developing severe forms of COVID-19 (48).

The significance of documenting the expression of these cytokines in outpatient gains notable relevance as it provides a more thorough understanding of the immune response, particularly from a hormonal standpoint. In this context, delving deeper into the unique patterns observed in outpatient is essential for the formulation of preventive and early disease management strategies. In the fascinating interplay of viral, hormonal, and immunological responses to COVID-19, an intriguing pattern emerges: premenopausal women exhibit a more robust expression of ACE2 and ERα, highlighting their uniqueness compared to postmenopausal women and affected men. This revelation suggests a richer and more complex network of interconnections than previously conceived in outpatients. These findings, more than mere discoveries, open doors to personalized strategies and treatments, while also inviting deep exploration into the fascinating intricacies of these interactions in different profiles of COVID-19 patients.

## 5 Conclusion

Our findings underscore the critical role of hormonal factors and cytokines in the immune response to COVID-19, particularly in premenopausal women. The higher expression of MIP-1β, IL-8, MCP-1, GM-CSF, and IL-7 in this group suggests a more robust and effective immune response during the early stages of infection, potentially contributing to improved clinical outcomes. These differences, along with the increased expression of ACE2 and hormonal receptors such as ERα, highlight the importance of considering gender and hormonal status in the design of therapeutic strategies. Moreover, our data emphasize the necessity of expanding research in a larger outpatient population to provide a more comprehensive understanding of disease progression and early management. This approach not only holds promise for optimizing personalized care but also opens new avenues for targeted interventions in specific populations.

## Data Availability

The original contributions presented in the study are included in the article/supplementary material, further inquiries can be directed to the corresponding authors.
